# Zirconium-89 Labeled Antibodies: A New Tool for Molecular Imaging in Cancer Patients

**DOI:** 10.1155/2014/203601

**Published:** 2014-05-28

**Authors:** Floor C. J. van de Watering, Mark Rijpkema, Lars Perk, Ulrich Brinkmann, Wim J. G. Oyen, Otto C. Boerman

**Affiliations:** ^1^Department of Radiology and Nuclear Medicine, Radboud University Medical Center, Geert Grooteplein Zuid 10, 6525 GA, Nijmegen, The Netherlands; ^2^Radboud Translational Medicine B.V., Reinier Postlaan 2, 6525 GC, Nijmegen, The Netherlands; ^3^Roche Pharma Research & Early Development, Large Molecule Research, Nonnenwald 2, 82377 Penzberg, Germany

## Abstract

Antibody based positron emission tomography (immuno-PET) imaging is of increasing importance to visualize and characterize tumor lesions. Additionally, it can be used to identify patients who may benefit from a particular therapy and monitor the therapy outcome. In recent years the field is focused on ^89^Zr, a radiometal with near ideal physical and chemical properties for immuno-PET. In this review we will discuss the production of  ^89^Zr, the bioconjugation strategies, and applications in (pre-)clinical studies of  ^89^Zr-based immuno-PET in oncology. To date, ^89^Zr-based PET imaging has been investigated in a wide variety of cancer-related targets. Moreover, clinical studies have shown the feasibility for ^89^Zr-based immuno-PET to predict and monitor treatment, which could be used to tailor treatment for the individual patient. Further research should be directed towards the development of standardized and robust conjugation methods and improved chelators to minimize the amount of released Zr^4+^ from the antibodies. Additionally, further validation of the imaging method is required. The ongoing development of new ^89^Zr-labeled antibodies directed against novel tumor targets is expected to expand applications of  ^89^Zr-labeled immuno-PET to a valuable method in the medical imaging.

## 1. Introduction

Molecular biomarkers can be used to monitor, image, and measure biological processes at molecular or cellular level. Different types of biomarkers are known, including diagnostic, prognostic, and predictive biomarkers, or a combination of these [1]. Extensive research has been done on the development of molecular imaging biomarkers in the field of cancer. This has led to tools that can be used to visualize and characterize tumor lesions. An advantage of using molecular imaging agents is the noninvasive nature of these procedures, whereas in conventional methods a more invasive procedure is used (e.g., blood sample or biopsy). Various imaging modalities can be used for tumor visualization such as fluorescent imaging, magnetic resonance imaging (MRI) or radionuclide imaging with positron emission tomography (PET), or single photon emission computed tomography (SPECT). In most cases, the use of PET is preferred over SPECT since higher spatial resolution images can be obtained and images can be analyzed quantitatively more accurately with PET. Specific uptake of molecular biomarkers can be achieved using radiolabeled targeting agents such as antibodies, directed against tumor-associated antigens like epidermal growth factor receptor (EGFR) [2], human epidermal growth factor receptor 2 (HER2), and many others. The high specificity and affinity of radiolabeled antibodies make them attractive candidates as an imaging agent. For example, ^89^Zr-labeled anti-HER2 antibodies can be used to differentiate between HER2^+^ and HER2^−^ tumors [3], also appreciating intra- and intertumoral heterogeneity. An additional application of radiolabeled antibodies is to identify patients who may benefit from a particular therapy and monitor therapy outcome based on the level of tumor-associated antigen expression [4]. However, the relative slow pharmacokinetics of intact antibodies (*t*_1/2_ = 3-4 days) requires the use of radionuclides with long half-lives (e.g., ^111^In (2.8 days) for SPECT or ^89^Zr (3.3 days) and ^124^I (4.2 days) for PET [5]). For antibody based PET imaging (immune-PET) ^89^Zr has several advantages: ^89^Zr has a half-life of 78.4 h which matches the pharmacokinetics of antibodies and it has a relative low average positron energy of 395 keV, making it an ideal candidate for high resolution PET imaging of slow-accumulating biomolecules. In addition, ^89^Zr-based agents are safer to handle and more stable* in vivo* making them better candidates than ^124^I-based agents for clinical applications. Due to the numerous advantages of ^89^Zr-based immuno-PET, the field is progressing at a rapid and exciting pace. In this review, the potential of ^89^Zr-based immuno-PET in oncology will be reviewed. The production of ^89^Zr, the bioconjugation strategies, and applications in (pre-)clinical studies are discussed.

## 2. Radiochemical Properties of ^89^Zr

^89^Zr decays (half-life of 78.4 h) first via positron emission and electron capture to ^89 m^Y (half-life of 15.7 s) which in turn decays via gamma ray emission (909 keV) to the stable ^89^Y. With its relatively low energy positrons (average energy 395 keV) ^89^Zr provides high resolution PET images. In addition, the energy disparity between the photons (511 keV) and the gamma rays (909 keV) prevents the latter from interfering with the detection of 511 keV photons. In contrast, its halogen competitor, ^124^I, produces high energy photons of different energies (603 keV (63.0%), 1691 keV (10.9%), and 723 keV (10.4%) [6]) which may result in random and scatter coincidences and therefore in more background noise as compared to ^89^Zr. Hence, reconstruction of ^89^Zr-based PET scans is more straightforward to attain good image quality compared to ^124^I. Although ^89^Zr has many advantages over other PET radionuclides, some essential shielding requirements during transport and handling of ^89^Zr are needed (half-value layer of ^89^Zr in lead is roughly 10 mm). High energy and highly penetrating photons (909 keV) are emitted during ^89^Zr decay in high abundance.

## 3. Production of ^89^Zr

The first production of ^89^Zr was done by Link et al. [7] by a (p,n) nuclear reaction by bombarding ^89^Y on Y foil with 13 MeV protons [5]. The produced ^89^Zr needed several purification steps and was obtained in 80% yield with radionuclidic purity exceeding 99%. Nowadays, many medical centers are able to produce medical isotopes using low-energy cyclotrons that are capable of bombarding targets with protons of low energy (<20 MeV). Therefore, the most common route to produce ^89^Zr is via the ^89^Y (p, n) ^89^Zr reaction on commercially available ^89^Y target foils. The above route will in general result in high yields (94-95%) and high radionuclidic purities (>99%). Competing nuclear reaction, like (p, 2n) reactions, can result in small amounts radionuclidic byproducts, such as ^88^Zr and ^88^Y [8]. Several separation and purification techniques with variable outcomes are used including anion exchange, cation exchange, and solvent extraction [9–11]. For synthesizing such radiopharmaceuticals for patients, automated units for a clean, fast, safe, and reproducible radionuclide synthesis according to good manufacturing practice (GMP) are necessary. Several groups have designed and built automated systems for ^89^Zr [12, 13]. For example Wooten et al. [14] reported a custom-made system to safely and routinely produce ^89^Zr with high radionuclidic purity (>99.99%) and satisfactory effective specific activity (5–353 mCi*·μ*mol^−1^ (0.01%–0.88% of theoretical specific activity)) based on previous developments in separation and purification techniques [9–11, 15].

## 4. The Need for Efficient Chelators

**Figure 1:**
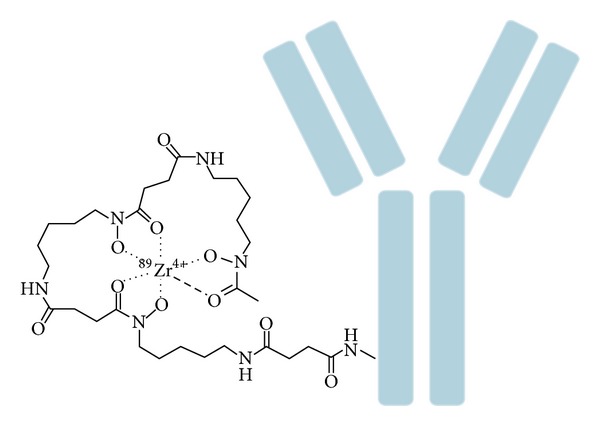
Schematic overview of ^89^Zr-labeled antibody using DFO as chelator.

The release of ^89^Zr^4+^ from the antibodies needs to be prevented, because the free radionuclide can accumulate in the mineral bone and can associate with plasma proteins. This leads to depositing significant doses to the bone marrow [16]. Therefore, an appropriate chelator system is necessary to minimize the disassociation of ^89^Zr from the antibodies. Over the years, several chelators have been used with different success, such as diethylenetriaminepentaacetic acid (DTPA), ethylenediaminetetraacetic acid (EDTA), 1,4,7,10-tetraacetic acid (DOTA), and desferoxamine (DFO) [17]. The stability of Zr-DOTA, Zr-DTPA, and Zr-EDTA was found to be limited. The thermodynamic stability of Zr-DTPA is slightly higher than that of Zr-EDTA, most likely because DTPA coordinatively saturates the Zr^4+^, while EDTA requires exogenous water molecules [18]. DFO is the most prominent chelator of Zr^4+^. DFO is a hexadentate siderophore containing three hydroxamate groups for chelating metals and a primary amine tail for conjugation to a biomolecule (Figure 1). Besides a zirconium chelator, it is a chelating agent for several other metal ions [19]. It demonstrated good stability, releasing less than 0.2% of Zr^4+^ after 24 h in serum [20] and after seven days in serum still less than 2% demetallation occurs [21]. Several proof-of-principle preclinical studies have been conducted using DFO to label antibodies with ^89^Zr; however, the* in vivo* stability of this complex remains an issue, because free ^89^Zr is observed in the bone dependent on the* in vivo* behavior of the antibody [22]. Several studies have attempted to improve the linkage between DFO and the antibody [9, 23], whereas others have focused on improving the chelate itself [24]. Eventually, a ligand that is both octadentate and oxygen-rich is believed to be the most stable Zr^4+^ chelator, since it would be able to incorporate all eight coordination sites of zirconium [22]. This novel high stability Zr^4+^ ligand would in theory minimize the uptake of liberated Zr^4+^ in the bone and other nontargeted tissues. To date, the design, synthesis, and the evaluation of such a Zr^4+^ chelate requires further research.

### 4.1. Conjugation of Antibodies with DFO

As DFO is currently the most promising chelator for ^89^Zr^4+^, conjugation of antibodies with DFO will be discussed here in detail (Figure 1). Several methods are available to conjugate DFO based on the reaction of an activated bifunctional chelator with a lysine or cysteine residue of the antibody. The different conjugation techniques do not only have different conjugation efficiencies, but also affect the biodistribution of the radiolabeled antibody [22].

The earliest reports on conjugation of DFO to bioactive molecules is based on the addition of thiols to the amino group of DFO [20]. In this approach DFO was modified by* N*-succinimidyl-S-acetylthioacetate (SATA), resulting in an S-acetyl-protected thiol derivatized form of chelator. In parallel, maleimide moieties were introduced in the antibody by the reaction with 4-(*N*-maleimidomethyl) cyclohexane carboxylic acid N-hydroxysuccinimide ester (SMCC). Next, the two formed compounds were combined and in the presence of hydroxylamine at physiological pH the DFO-antibody conjugate was formed. Following this early work, Verel et al. introduced a novel conjugation approach, which was based on an activated 2,3,5,6-tetrafluorophenol (TFP) chelate ester which can form a stable amide bond with the *ε*-amino-groups of the lysine residues of the monoclonal antibody (mAb) [9]. This laborious approach consisted of 5 steps which involve (i) the extension of DFO with succinyl anhydride, (ii) protection of side-reactions of the hydroxamate groups of the ligand by complexation with Fe^3+^, (iii) formation of activated TFP ester, (iv) conjugation of activated DFO-ester to the unmodified antibody, and (v) removal of Fe^3+^ from the chelator. Nowadays the most widely used method in preclinical ^89^Zr-based immuno-PET uses the conjugation strategy with* N*-succinimidyl-DFO, which addresses *ε*-amino groups of lysine side chains [22, 25–30]. Since the Zr^4+^ field is rapidly growing and becoming more mainstream in the clinical setting, simple methods for DFO conjugation preferably using commercial available starting materials are essential. Perk et al. introduced a simplified method using a commercially available* p*-isothiocyanatobenzyl-DFO (DFO-Bz-NCS) chelate, which can be directly attached to the *ε*-aminogroups of the lysine residues of an antibody by forming a stable thiourea linkage [23]. Despite the fact that this method is simpler than the* N*-succinimidyl-DFO chemistry, it requires more expertise mainly because of the limited water solubility of the chelator precursor.

A limitation of the conjugation of DFO to antibodies is compromised immunoreactivity, because the chelator may interfere with the antigen-binding domain of the antibody, especially if there are lysines in or close to the complementarity determining regions of the antibody. To overcome these limitations site-specific strategies using engineered cysteine residues can be used in combination with thiol-reactive DFO derivatives such as bromoacetamido-desferrioxamine (DFO-Bac), iodoacetamido-desferrioxamine (DFO-Iac), and maleimidocyclohexyl-desferrioxamine (DFO-CHX-Mal) [31]. The radiolabeled antibodies using these thiol-reactive DFO derivatives were stable and showed similar characteristics as the lysine-linked complexes. Remarkably, no significant difference was observed between the immunoreactivity of the site-specific complex and the lysine-linked complexes.

Another novel conjugation approach for effective labeling of ^89^Zr to antibodies is the use of click chemistry between an acetylene group and an azide. This approach might not significantly improve the targeting of tumors compared with DFO-based conjugation strategies; however, with this approach it is possible to fully tailor the constructs. Furthermore, the modular system can be used for direct comparison of bioconjugates with different radiometals as the the chelator-modified antibodies are synthesized using identical ligation conditions resulting in similar immunoreactivity and chelator/antibody ratios [32]. Several studies have been reported on bioorthogonal click chemistry [32], Staudinger ligation [33], or catalyst-free click chemistry [34]. The click chemistry as specialized conjugation method is expected to expand the scope of ^89^Zr-based PET.

## 5. Preclinical Studies with ^89^Zr 

Over the last years several ^89^Zr-labeled antibodies directed against different tumor types have been evaluated in preclinical studies (e.g., [9, 18, 22, 35, 36]; see Table 1). Here these developments of ^89^Zr-labeled antibodies in preclinical studies will be discussed based on their tumor target.

### 5.1. Targeting CD20

The glycosylated phosphoprotein, CD20, is expressed on the surface of B-cell lymphomas, hairy leukemia, B-cell chronic lymphocytic leukemia, and melanoma cells. ^89^Zr-labeled antibodies directed against CD20 might be useful to measure and monitor the therapeutic effect of non-Hodgkin's lymphoma (NHL) therapy [18, 37]. The ^89^Zr-Desferrioxamine-rituximab, an antibody directed against CD20, specifically targeted the human CD20 antigen in a humanized CD20-expressing transgenic mouse model (huCD20TM). ^90^Y-labeled anti-CD20 mAb ibritumomab tiuxetan (Zevalin) is approved for treatment of patients with relapsed and refractory NHL. In a pilot study, ^89^Zr-labeled ibritumomab tiuxetan was shown to have a nearly identical biodistribution compared to ^90^Y-labeled counterpart [18]. This indicated that a scout scan with ^89^Zr-ibritumomab immuno-PET can be used to assess, predict, and quantify the biodistribution of  ^90^Y-ibritumomab tiuxetan.

### 5.2. Targeting CD44

The cell-surface glycoprotein, CD44, is involved in many biological processes including adhesion of cells to extracellular matrix proteins, lymphocyte-endothelial cell interactions, metastasis formation, migration of cells, and T cell activation/adherence [38]. The v6 splice variant of CD44 is involved in tumorigenesis, tumor cell invasion, and metastasis and is expressed preferentially in squamous cell carcinomas [39]. Preclinical studies using ^89^Zr-labeled anti-CD44v6 chimeric monoclonal antibody cU36 demonstrated that the tracer was able to detect small tumors in nude mice with HNSCC xenografts [9, 40]. In addition, it was reported that ^89^Zr-cU36 PET imaging was a suitable candidate for scouting of therapeutic doses of ^90^Y-cU36 [40, 41]. Recently, evaluation of ^89^Zr-RG7356, an antibody directed against the constant part of CD44, was performed in mice bearing tumor xenografts with different levels of CD44 expression and RG7356 responsiveness, namely, MDA-MB-231 (CD44+, responsive), PL45 (CD44+, nonresponsive), and HepG2 (CD44−, nonresponsive) [42]. ^89^Zr-RG7356 selectively targeted CD44+ responsive and nonresponsive tumors in mice. ^89^Zr-RG7356 whole body immuno-PET in healthy cynomolgus monkeys revealed antibody uptake in spleen, salivary gland, and bone marrow, which might be related to the expression of CD44 in these organs. The ^89^Zr-RG7356 uptake in the normal organs decreased with increasing dose of unlabeled RG7356, indicating saturable targeting of CD44 in these animals.

### 5.3. Targeting EGFR

The epidermal growth factor receptor (EGFR) is a member of the ErbB family. It plays a crucial role in differentiation, proliferation, and survival of many different tumor types, including breast, lung bladder, and colon carcinoma [2]. The overexpression of EGFR is associated with more aggressive tumors and poor prognosis due to the resistance of treatment [43, 44]. Many mAbs have been developed to inhibit the EGFR activation [2]. A well-known example is cetuximab (Erbitux), a chimeric IgG, which upon binding to the ligand-binding domain induces internalization of EGFR and thereby blocking downstream signalling [45, 46]. Several studies showed tumor regression upon treatment with cetuximab [47–50].

^89^Zr-labeled cetuximab was evaluated for scouting the biodistribution of  ^90^Y- and ^177^Lu-cetuximab in tumor bearing mouse and thus potentially allowing the estimation of the radiation dose delivered to tumors and normal tissues during radioimmunotherapy with ^90^Y- and ^177^Lu-cetuximab [51]. It was reported that the ^89^Zr-immuno-PET could be used for* in vivo* scouting of  ^90^Y- and ^177^Lu-labeled mAbs. However, an increased bone uptake of ^89^Zr-cetuximab, compared with ^90^Y- and ^177^Lu labeled cetuximab, was observed indicating that ^89^Zr is more efficiently incorporated in the bone compared to the other radiometals (^90^Y- and ^177^Lu). Therefore estimating bone marrow doses based on ^89^Zr-bone uptake is not straightforward. Another study investigated the relation between the* in vivo *expression of EGFR and the tumor uptake of ^89^Zr-cetuximab [52]. In this study no clear-cut relationship was found, suggesting that apart from antigen expression other parameters determine the tumor uptake of ^89^Zr-cetuximab.

Another approved mAb to inhibit the EGFR signalling is panitumumab. It was the first recombinant human monoclonal antibody (IgG2) approved by the FDA for the treatment of patients with EGFR-expressing metastatic colorectal cancer (mCRC) [53]. In several studies the use of panitumumab for noninvasive,* in vivo* imaging of HER1 expression in tumors is reported [54–58]. The use of  ^89^Zr-panitumumab for immuno-PET of HER1 expression was recently evaluated in a direct comparison with ^111^In-panitumumab. The organ biodistribution between ^111^In- and ^89^Zr-panitumumab was almost identical [55]. In addition, the targeting of ^89^Zr-panitumumab correlated well with the HER1 expression. Recently, a standardized and straightforward stepwise ~5 h production method was reported for the production of clinical-grade ^89^Zr-panitumumab [59]. In this method clinical-grade panitumumab is conjugated with DFO chelate and subsequently radiolabeled with ^89^Zr resulting in high yields (>70%) and high radiochemical purity (>98%).

### 5.4. Targeting HER2

**Figure 2:**
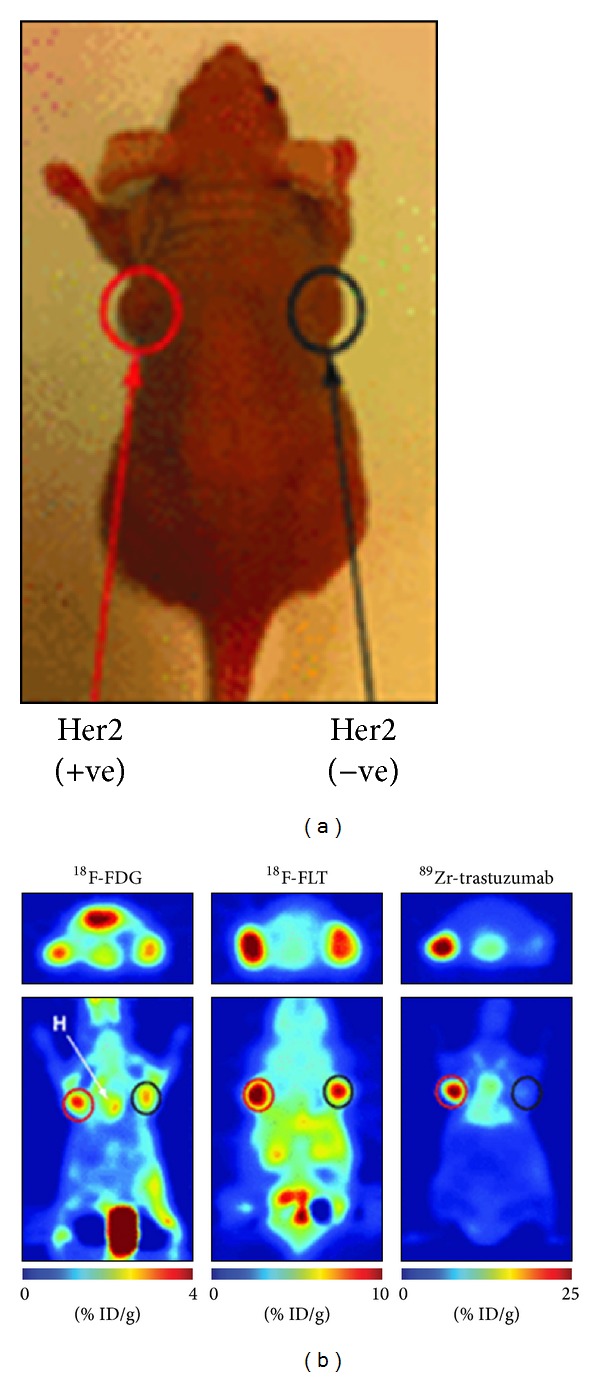
Specificity of ^89^Zr-trastuzumab for HER2-positive tumors. Coronal ^89^Zr-trastuzumab, ^18^F-FDG, and ^18^F-FLT PET images of athymic nude mice bearing subcutaneous HER2-positive NCI-N87 (left) and HER2-negative MKN-74 (right) are shown. ROIs (%ID/g) for ^89^Zr-trastuzumab, ^18^F-FDG, and ^18^F-FLT are indicated. +ve = positive; −ve = negative. This research was originally published in [3]. © by the Society of Nuclear Medicine and Molecular Imaging, Inc.

Human epidermal growth factor receptor 2 (HER2) is another member of the ErbB family. It is involved in angiogenesis, differentiation, metastasis, proliferation, and cell survival upon heterodimerization with other members of the EGF receptor family [60]. HER2 overexpression is found in many types of tumors including breast and ovarian cancer. The FDA approved anti-HER2 mAb trastuzumab (Herceptin, Genentech, CA, USA) to be used for the treatment of HER2 positive breast tumors, since it blocks the HER2 activation [60]. The efficacy of the treatment is dependent on the HER2 expression level. The HER2 expression level in a tumor is not static and may vary over time [60]. In addition, the HER2 expression is found to be different between the primary lesion and the distant metastatic lesions in the same patient. Noninvasive* in vivo* imaging to visualize HER2 expressing using radiolabeled trastuzumab has been extensively investigated [29, 30, 61]. PET imaging using ^89^Zr-trastuzumab has been performed in different murine tumor models and accumulation of the tracer was found to be HER2 specific [29, 30, 61]. For example, the tumor uptake of ^89^Zr-trastuzumab in nude mice with a subcutaneous human ovarian cancer xenografts (SK-OV-3) was high (~30% ID/g) and the biodistribution was similar to that of ^111^In-trastuzumab [29]. Recently, the specificity of ^89^Zr-trastuzumab, ^18^F-FDG, and ^18^F-FLT PET for HER2-positive gastric cancer was evaluated ([3]; Figure 2). The study revealed a high specific uptake of ^89^Zr-trastuzumab in HER2-positive tumors, whereas ^18^F-FDG and ^18^F-FLT PET were unable to differentiate between HER2-positive and HER2-negative tumors. In addition, ^89^Zr-trastuzumab was used to quantitatively determine the HER2 expression level after treatment. For example, after treatment with a heat shock protein 90 (hsp90) inhibitor a significant decrease in HER2 expression could be measured based on the ^89^Zr-trastuzumab tumor targeting [30, 62]. A combination treatment of hsp90 inhibitor 17AAG and the EGFR/HER2 tyrosine kinase inhibitor, lapatinib, revealed an even stronger reduction of the HER2 expression levels using ^89^Zr-Trastuzumab-F(ab′)_2_ fragment as probe [63]. Additionally, the biological effect of afatinib, an EGFR/HER2/HER4 inhibitor, in a HER2-positive gastric xenograft models was evaluated [3]. In this model the uptake of ^18^F-FDG did not change after afatinib therapy, whereas a decrease in ^89^Zr-trastuzumab uptake was observed upon treatment. The lower uptake of the ^89^Zr-trastuzumab correlated with the decreased HER2 expression as determined by immunoblots and immunohistochemistry. Thus, ^89^Zr-trastuzumab PET might be useful for the characterization, treatment planning, and treatment monitoring of HER-2 positive cancers.

### 5.5. Targeting VEGF

Vascular endothelial growth factor (VEGF) is a proangiogenic factor in both normal tissues and in tumors. The overexpression of VEGF and its receptors (VEGFR) are associated with poor prognosis [64]. The humanized anti-VEGF mAb, bevacizumab (Avastin, Genentech/Hoffmann-La Roche), is capable of blocking angiogenesis by depleting VEGF and thereby preventing its binding to the VEGFR. This neutralizes VEGF actions (see, e.g., [65–72]). A direct comparison between ^89^Zr-bevacizumab and an irrelevant ^89^Zr-labeled IgG revealed a significantly higher tumor uptake of ^89^Zr-bevacizumab in nude mice with human ovarian SK-OV-3 tumors [73]. Besides using ^89^Zr-bevacizumab as PET tracer for noninvasive* in vivo* imaging of VEGF expression in the tumor microenvironment, potentially it can also be used to predict or monitor an antiangiogenic response. For example, hsp90 is crucial player in VEGF transcription and can be used to treat ovarian tumors. In nude mice with a subcutaneous human ovarian cancer xenografts (A2780), uptake of ^89^Zr-bevacizumab in the tumors correlated with the therapeutic effect of the hsp90 inhibitor, NVP-AUY922, [74]. In another study the effect of the mTOR inhibitor, everolimus, on the VEGF production was evaluated [75]. Everolimus treatment caused decreased ^89^Zr-bevacizumab uptake in subcutaneous A2780 human ovarian tumor. The results were in line with the lower VEGF-A protein levels in tumor lysates of treated versus untreated tumors. These results indicate ^89^Zr-bevacizumab can be used to monitor tumor VEGF-A levels as an early biomarker of the antiangiogenic effect of mTOR inhibitor treatment.

^89^Zr-labeled ranibizumab, a monoclonal antibody fragment (Fab) derivative of bevacizumab, was used to detect and monitor the early antiangiogenic response to treatment with sunitinib, a VEGFR tyrosine kinase inhibitor, in nude mice bearing a subcutaneous A2780 human ovarian tumor or Colo205 human colon cancer xenografts. ^89^Zr-ranibizumab PET matched better with the observed results obtained by histology, immunohistochemistry, and tumor proliferation and vascularization assays, than ^18^F-FDG PET and ^15^O-water PET. Since ranibizumab has a serum half-life of only 2 to 6 hours, rapid and sequential follow-up PET scans are feasible with ^89^Zr-ranibizumab [76]. Therefore, in contrast to ^89^Zr-bevacizumab, ^89^Zr-ranibizumab can be used for imaging of rapid dynamic alterations in VEGF response in tumors.

### 5.6. Targeting PIGF

The clinical benefits of angiogenesis inhibitors can be compromised by the upregulation of proangiogenic factors such as the placental growth factor (PIGF). PIGF, a VEGF homolog, is expressed in low levels in normal tissue and can be overexpressed in tumor cells. PIGF contributes to angiogenesis in pregnancy, wound healing, ischemic conditions, and tumor growth [77, 78]. PIGF inhibitors are able to reduce the angiogenesis and tumor cell motility. The antitumor activity of a humanized mAb directed against PIGF-1 and PIGF-2, RO5323441, in human tumor xenograft models has been reported [79]. To further explore and validate the use of RO5323441, the tumor and normal tissue uptake of ^89^Zr-RO5323441 at different time points was evaluated in mice bearing human PlGF-expressing Huh7 hepatocellular cancer xenografts. Tumor accumulation of ^89^Zr-RO5323441 was specific and time- and dose-dependent.

### 5.7. Targeting PSMA

Prostate-specific membrane antigen (PSMA) is a transmembrane glycoprotein which is associated with increased tumor progression, development of castration resistance, and/or resistance to hormone-based treatments [80–82]. PMSA is expressed in a limited range of normal tissues including benign prostatic epithelium, renal proximal tubule, small bowel, and the brain; however, the expression level is 2 to 3 times lower than in prostate cancer specimens [83]. ^89^Zr-labeled anti-PSMA mAb, J591, was able to differentiate between subcutaneous PSMA positive and negative tumors in athymic nude mice [21], making it a potential target for clinical noninvasive identification and quantification of PSMA-positive tumors.

### 5.8. Targeting CD147

**Figure 3:**
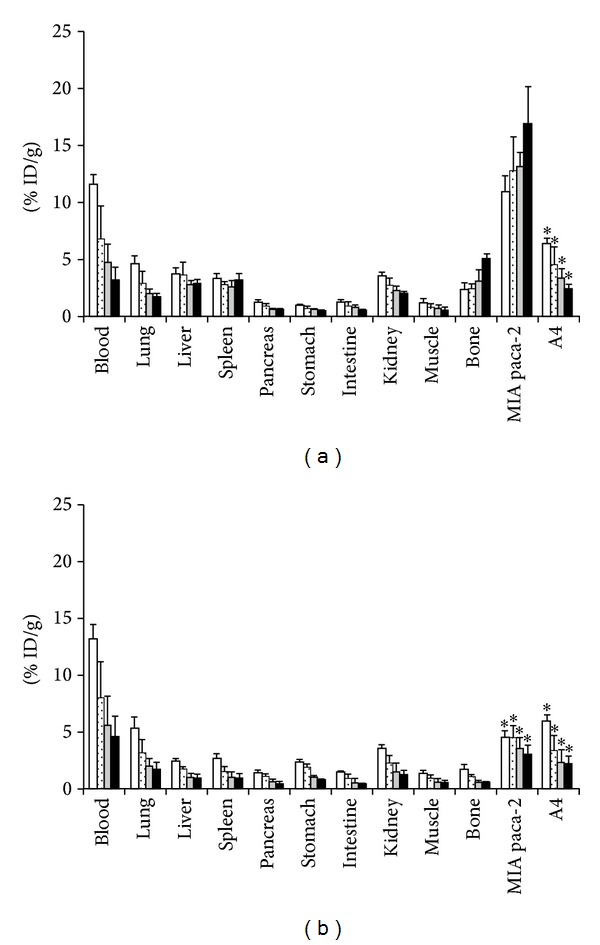
*In vivo* biodistribution experiments in nude mice bearing MIA PaCa-2 and A4 xenografts of radiolabeled anti-CD147 antibody 059-053. Samples were collected and weighted, and radioactivity was measured at days 1 (white bars), 2 (dot bars), 4 (gray bars), and 6 (black bars) after intravenous injection of 37 kBq each of ^89^Zr-059-053 (a) and ^125^I-059-053 (b). Data are expressed as mean ± SD (*n* = 5). *P < 0.01 versus ^89^Zr-059-053 tumor uptake at each time point analyzed by ANOVA with the Student-Newman-Keuls method multiple comparison test. This research was originally published in [84].

CD147, a member of the immunoglobulin superfamily, is involved in many physiological functions including embryo implantation, early stage neural network formation, and spermatogenesis [85]. Overexpression of CD147 is found in many types of cancer including pancreatic cancer and induces expression of matrix metalloproteinases (MMPs) and VEGF [86, 87]. Several (pre-)clinical studies have been performed using anti-CD147 antibodies to inhibit the actions of CD147 and revealed a reduction in proliferation, invasion and metastasis of tumors [88–90]. Almost 90% of the pancreatic cancers have high CD147 expression levels [86]. Sugyo et al. evaluated the CD147 expression in four pancreatic cancer cell lines (MIA Paca-2, PANC-1, BxPC-3, and AsPC-1) using the human ^125^I-, ^67^Ga-, or ^89^Zr-labeled anti-CD147 mAb (059-053) [84]. Additionally, the* in vivo* CD147 expression was evaluated using ^125^I- or ^89^Zr-labeled 059-053 in mice with s.c. and orthotopic MIA Paca-2 and A4 (non-CD147-expressing) tumors. The biodistribution data revealed significantly higher tumor uptake of ^89^Zr-059-053 in MIA Paca-2 tumors than in the A4 tumors (Figure 3). PET/CT imaging demonstrated that orthotopic MIA Paca-2 tumors could be visualized with ^89^Zr-059-053 PET. High expression of CD147 is not only restricted to pancreatic cancer, but is also found in other types of cancer including bladder, breast, colorectal, cervical, liver, and ovarian cancer [84–86]. Therefore, ^89^Zr-059-053 might also be applied in patients with these cancer types.

### 5.9. Targeting CAIX

Hypoxia in tumors is associated with a poor prognosis in many tumor types since it is associated with resistance to radiotherapy and chemotherapy. In many tumor types carbonic anhydrase IX (CAIX) has been validated as an intrinsic hypoxia-related cell marker [91]. Using antibodies directed against CAIX it is possible to select patients for hypoxia-targeting or -modifying treatment combined with radiotherapy. For example, it is possible to visualize tumor hypoxia in mice bearing s.c. SCCNij3 head and neck squamous cell carcinomas using ^89^Zr-cG250-F(ab′)_2_, an anti-CAIX antibody fragment [92]. In a direct comparison, the tumor uptake of mAb ^89^Zr-cG250 in mice with CAIX-expressing clear cell renal cell carcinoma (ccRCC) xenografts (NU-12) was significantly higher compared to that of ^124^I-cG250 [93]. This indicates that PET imaging of ccRCC tumors with ^89^Zr-cG250 could be more sensitive than ^124^I-cG250-PET. CAIX targeted ^89^Zr-PET imaging is a candidate for imaging hypoxia in different types of tumors and deserves further exploration.

### 5.10. Targeting IGF-1R

The insulin like growth factor 1 receptor (IGF-1R) is a transmembrane receptor expressed in many human cancers, including in ~35% of all triple-negative breast carcinomas. It is involved in the proliferation, apoptosis, angiogenesis, and tumor invasion. Heskamp et al. reported excellent tracer uptake of ^111^In-R1507 and ^89^Zr-R1507, a human mAb directed against IGF-1R, in mice with s.c. SUM149 triple-negative breast cancer xenografts [94]. This suggests that the use of ^89^Zr-R1507 in patient selection of IGF-1R-targeted therapy is possible.

### 5.11. Targeting Met

The expression of hepatocyte growth factor receptor tyrosine kinase (Met) was measured by PET using ^76^Br or ^89^Zr-labeled-onartuzumab, a mAb against Met [95]. Both tracers specifically targeted Met; however, at later time points a higher tumor uptake was observed with ^89^Zr-Onartuzumab. This suggests that ^89^Zr-onartuzumab is the preferred tracer to identify Met expression in cancer patients and possibly to predict and monitor the treatment with Met-targeted therapeutics. In another study, the potential of immune-PET using ^89^Zr (residualising radionuclide) or ^124^I-labeled (non-residualising radionuclide) anti-Met mAb DN30 was evaluated in mice with s.c. GLT-16 (high Met expression) and FaDu (low Met expression) tumors [96]. The biodistribution data revealed significantly higher tumor uptake of ^89^Zr-DN30 than ^124^I-DN30 in GTL-16 tumor-bearing mice. Similar blood levels were found indicating that DN30 is internalized. ^89^Zr-DN30 immuno-PET imaging was able to visualize small tumor lesions with a higher ^89^Zr tumor uptake in GTL-16 than FaDu tumor-bearing mice. Additionally, the correlation was high for PET-image-derived ^89^Zr tumor uptake and the* ex vivo*-assessed ^89^Zr tumor uptake. This indicates that ^89^Zr-labeled immuno-PET is an attractive method to evaluate Met-targeted therapeutics.

### 5.12. Targeting GPC3

The glypican-3 (GPC3) is a hepatocellular-specific cell surface proteoglycan overexpressed in most hepatocellular carcinomas (HCC). Sham et al. reported excellent tracer uptake of ^89^Zr-*α*GPC3, a mAb directed against GPC3, in mice with GPC3-expressing HepG2 liver tumors [97]. This suggests that the use of ^89^Zr-*α*GPC3 to image HCC in the liver is possible.

## 6. Clinical Translation of ^89^Zr Immuno-PET

The ^89^Zr-labeled antibodies against the targets mentioned above all show promising results for clinical translation. To date, several clinical investigations using ^89^Zr-labeled antibody constructs have been reported [1, 22, 98]. Here these recent clinical studies will be discussed.

### 6.1. ^89^Zr-Labeled cU36

The first clinical trial using the ^89^Zr-cU36 PET to target CD44 expressing tumors showed that the tracer was able to detect primary tumors as well as metastases in the neck region with similar sensitivity as computed tomography (CT) and magnetic resonance imaging (MRI) [99]. The results are promising, although several issues remain to be addressed. In the clinical study micrometastases were missed with ^89^Zr-cU36 PET, so immuno-PET may be less suited as a staging tool, but more suitable to characterize tumors. Moreover, 2 out of the 20 patients developed antibodies against the chimeric cU36 antibody (HACA), which may hinder repetitive imaging procedures.

### 6.2. ^89^Zr-Ibritumomab

A clinical prospective study was conducted to evaluate the biodistribution and radiation dosimetry of CD20-targeting ^90^Y-ibritumomab tiuxetan using ^89^Zr-ibritumomab tiuxetan [100]. Patients with relapsed or refractory aggressive B-cell (CD20-positive) NHL underwent a PET scan at 1, 72 and 144 h after injection of 70 MBq ^89^Zr-ibritumomab tiuxetan and again 2 weeks later after coinjection of 15 MBq/kg or 30 MBq/kg ^90^Y-ibritumomab tiuxetan. The results revealed that simultaneous therapy of ^90^Y-ibritumomab tiuxetan did not affect the biodistribution of ^89^Zr-ibritumomab. A second aim of the study was to estimate the radiation doses during radioimmunotherapy with ^90^Y-ibritumomab tiuxetan based on ^89^Zr-ibritumomab PET. The highest ^90^Y absorbed dose was observed in liver (3.2 ± 1.8 mGy/MBq) followed by the spleen (2.9 ± 0.7 mGy/MBq). Additionally, the correlation was high for standardized uptake value (SUV) of ^89^Zr-ibritumomab tiuxetan and absorbed dose of ^90^Y-ibritumomab tiuxetan in the liver at 72 h p.i. and 144 h p.i. This suggests that in the future a single ^89^Zr-ibritumomab tiuxetan PET scan is sufficient to optimize the administered amount of ^90^Y-ibritumomab tiuxetan RIT for individual patients

### 6.3. ^89^Zr-Trastuzumab

**Figure 4:**
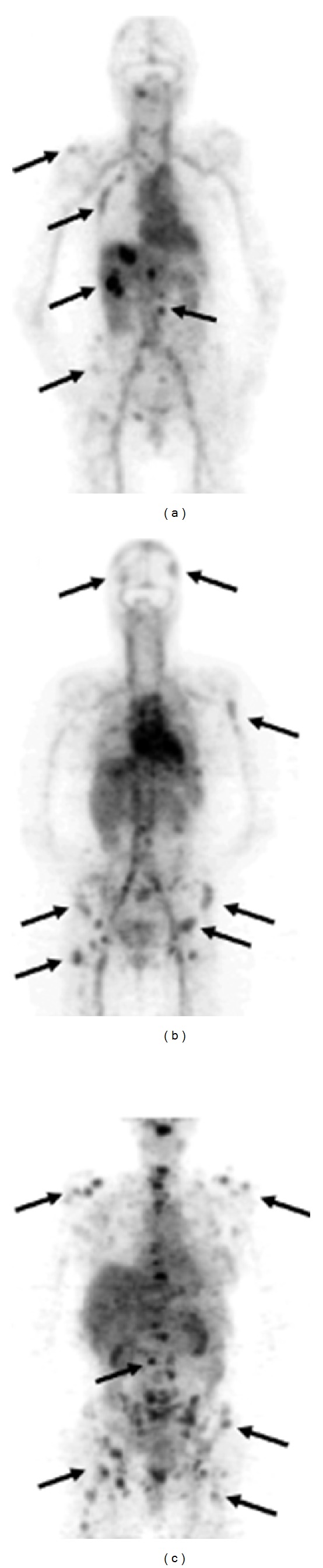
Examples of ^89^Zr-trastuzumab uptake 5 days after the injection: (a) a patient with liver and bone metastases and ((b) and (c)) two patients with multiple bone metastases. A number of lesions have been specifically indicated by arrows. This research was originally published in [101].

In 2010, the first-in-man report of ^89^Zr-trastuzumab for imaging of HER2-positive lesions in patients with metastatic breast cancer was published [101]. 14 Patients were included in the study that either received 10 (*n* = 2) or 50 (*n* = 5) mg ^89^Zr-trastuzumab if trastuzumab-naïve and 10 mg ^89^Zr-trastuzumab (*n* = 7) if on trastuzumab treatment (37 MBq ^89^Zr-trastuzumab). Per patient at least two PET scans were acquired between day 2 and day 5 after injection of ^89^Zr-trastuzumab. The trastuzumab-naïve patients required a 50 mg dose for effective imaging whereas 10 mg was sufficient in the trastuzumab-treated patients. A higher dose in the trastuzumab-naïve patients was required as an increased ^89^Zr-trastuzumab clearance was observed at lower doses due to presence of extracellular domains of the HER2 receptor in the circulation [102]. After binding of ^89^Zr-trastuzumab to these extracellular domains, the immune complex was cleared by the liver and excreted in the intestines. In patients treated with trastuzumab at the time of injection, higher doses of ^89^Zr-trastuzumab did not improve imaging since complex formation was minimal. Overall, the uptake of ^89^Zr-trastuzumab in the tumor lesions was high. The best time to assess tumor uptake was 4 to 5 days after injection of ^89^Zr-trastuzumab (Figure 4). All the known and even some unknown lesions were detected with PET. Of interest, metastatic brain lesions were detected in several patients, despite the fact that trastuzumab cannot penetrate the blood-brain barrier. This is probably because the blood-brain barrier in patients with brain metastasis is disrupted allowing ^89^Zr-trastuzumab to pass. In this study HER2 overexpressing lesions could be distinguished from non-HER2 expressing lesions. These data indicate the potential use of ^89^Zr-trastuzumab to improve the diagnosis of patients with HER2-positive breast cancer especially when lesions are inaccessible for biopsy.

### 6.4. ^89^Zr-Bevacizumab

**Figure 5:**
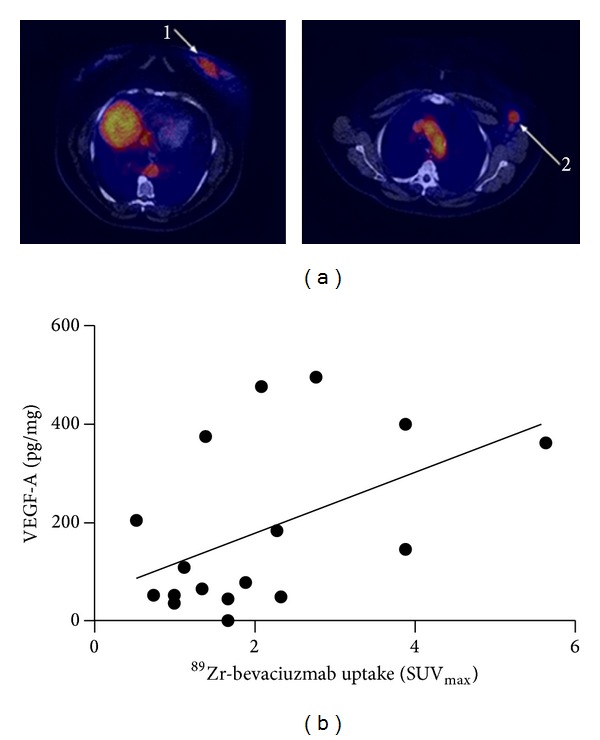
(a) Axial slices of ^89^Zr-bevacizumab PET from patient with primary breast tumor (1) and lymph node metastasis (2). (b) Correlation between ^89^Zr-bevacizumab tumor uptake (*x*-axis) and tumor VEGF-A (*y*-axis) levels as measured by ELISA (Pearson *r* = 0.49, *P* = 0.04). This research was originally published in [103]. © by the Society of Nuclear Medicine and Molecular Imaging, Inc.

Recently, a clinical study was performed to assess the use of ^89^Zr-bevacizumab for the visualization of VEGF-A in primary breast cancer [103]. In 23 patients, 26 tumors were detected by conventional imaging modalities mammography (*n* = 22), ultrasound (*n* = 25), or MRI (*n* = 1). Prior to surgery and 4 days p.i. of 37 MBq of ^89^Zr-bevacizumab the patients underwent a PET/CT scan of the breasts and the axillary regions (Figure 5(a)). 25 of the 26 breast cancer nodules (96.1%) were detected using ^89^Zr-bevacizumab. Also, a correlation between the VEGF-A protein level in the tumors observed as measured by VEGF-A ELISA and the tumor uptake ^89^Zr-bevacizumab was found (Figure 5(b)). This study provides evidence that ^89^Zr-bevacizumab might be a potential candidate for the classification of breast tumors and to predict and monitor the effect of VEGF-A targeted therapies.

## 7. Conclusions 

Clinical studies revealed that the use of ^89^Zr-based immuno-PET results in high spatial resolution images with high tumor uptake and a good signal to noise ratio. Therefore, the use of ^89^Zr-labeled antibodies is very promising for noninvasive visualization of tumor-associated antigens before, during, and after therapy. This makes ^89^Zr-based immuno-PET an excellent imaging modality to predict and monitor treatment and to tailor treatment for individual patients. However, to fully integrate ^89^Zr-based immuno-PET in the clinic several hurdles still need to be overcome. For example, standardized and robust methods for stable conjugation of DFO to antibodies should become available to obtain clinical-grade conjugates. In addition, research should focus on the development of improved chelators to minimize the amount of liberated Zr^4+^. Although some direct comparison studies between ^89^Zr-based immuno-PET and immuno-PET using other PET isotopes have been performed, and supplementary quantitative and comprehensive comparison studies are needed to evaluate the value of ^89^Zr-based immuno-PET. Additionally, the radiation dose for patients undergoing a ^89^Zr-based immuno-PET (75 MBq of ^89^Zr-cmAb U36) was found to result in a mean effective dose of 0.53 to 0.66 mSv/MBq [104] which is significantly higher compared to the mean effective dose of clinically used ^111^In- and ^99^Tcm-based tracers (^111^In-IgG (75 MBq) was 0.25 mSv/MBq and ^99^Tcm-IgG (750 MBq) was mu Sv/MBq) [105]. The high radiation dose for patients will limit repeated application of ^89^Zr-based immuno-PET [104]. However, introducing new PET/CT scanners to allow better-quality immuno-PET images to be obtained with a lower ^89^Zr radioactivity (37 MBq) dose have reduced the radiation dose [102, 103]. Furthermore, research is focusing on combining ^89^Zr-based immuno-PET with other imaging modalities. For example, the use of ^89^Zr-immuno-PET in combination with near-infrared fluorescence (NIRF) imaging has been reported by several groups [106–108]. The ongoing development of new ^89^Zr-labeled antibodies directed against novel tumor targets is believed to rapidly expand applications of ^89^Zr-labeled immuno-PET to a valuable method in the medical imaging.

## Figures and Tables

**Table 1:** Overview of the described preclinical and clinical studies using ^89^Zr-labeled antibodies.

Target	Type of tumor	Targeting vector
CD147	Pancreas	059-053
CD20*	Non-Hodgkin's lymphoma	ibritumomab tiuxetan
CD44v6*	Head and neck squamous cell carcinoma	cmAb U36
EGFR	Multiple	Cetuximab
EGP-1	Prostate	hRS7
GPC3	Liver	*α*GPC3
HER1	Colorectal	Panitumunmab
HER2*	Breast and ovarian	Trastuzumab
IGF-1R	Triple negative breast cancer	R1507
MET	Head and neck squamous cell carcinoma and gastric	DN30
MN/CA IX	Renal cell carcinoma	cG250
PSMA	Prostate	7E11
PIGF	Liver	RO5323441
VEGF*	Breast, head, and neck squamous cell carcinoma and ovarian	Bevacizumab

*Targets evaluated in clinical studies.
